# Epidemiologic and Virologic Characteristics of Influenza in Lao PDR, 2016–2023

**DOI:** 10.1111/irv.13353

**Published:** 2024-08-05

**Authors:** Natalie Wodniak, KeoOudomphone Vilivong, Bouaphanh Khamphaphongphane, Bounthanom Sengkeopraseuth, Virasack Somoulay, May Chiew, Pakapak Ketmayoon, Melissa Jiao, Sonesavanh Phimmasine, Kim Carmela Co, Phetdavanh Leuangvilay, Satoko Otsu, Viengphone Khanthamaly, Phayvanh Keopaseuth, William W. Davis, Martha P. Montgomery, Phonepadith Xangsayyarath

**Affiliations:** ^1^ Thailand Ministry of Public Health‐U.S. Centers for Disease Control and Prevention Collaboration Nonthaburi Thailand; ^2^ National Center for Laboratory and Epidemiology Vientiane Lao People's Democratic Republic; ^3^ WHO Health Emergencies Programme World Health Organization Vientiane Lao People's Democratic Republic; ^4^ U.S. Centers for Disease Control and Prevention Collaboration‐Laos Vientiane Lao People's Democratic Republic; ^5^ Influenza Division National Center for Immunization and Respiratory Diseases, U.S. Centers for Disease Control and Prevention Atlanta Georgia USA; ^6^ Ministry of Health Vientiane Lao People's Democratic Republic

**Keywords:** epidemiology, hospitalization, human influenza, reverse transcriptase polymerase chain reaction, sentinel surveillance, vaccination

## Abstract

**Background:**

Influenza sentinel surveillance in Lao PDR is used to inform seasonal vaccination programs. This analysis reviews epidemiologic and virologic characteristics of influenza virus infection over 8 years, before and after emergence of SARS‐CoV‐2.

**Methods:**

Data collected for ILI and SARI surveillance during January 2016 through December 2023 were analyzed from nine hospitals. Respiratory specimens from ILI and SARI cases were tested by reverse transcriptase polymerase chain reaction to determine influenza positivity and subtype and lineage. Aggregate counts of outpatient visits and hospitalizations were collected from hospital logbooks. Epidemiologic trends of influenza activity were described, and the proportional contribution of influenza‐associated ILI and SARI to outpatient and inpatient loads was estimated.

**Results:**

Influenza was detected year‐round with positivity peaking during September through January and occurring in most years approximately 1 month earlier in the south than the north. After decreasing in 2 years following the emergence of SARS‐CoV‐2, influenza positivity increased in 2022 and resumed its typical temporal trend. Influenza‐associated ILI contribution to outpatient visits was highest among children ages 5–14 years (3.0% of all outpatient visits in 2023), and influenza‐associated SARI contribution to inpatient hospitalizations was highest among children ages 2–4 years (2.2% of all hospitalizations in 2023).

**Conclusions:**

Influenza surveillance in Lao PDR provides clinicians and public health authorities with information on geographic and temporal patterns of influenza transmission. Influenza surveillance data support current vaccination timing and recommendations to vaccinate certain populations, especially young children.

## 1 Introduction

In Lao People's Democratic Republic (Lao PDR), surveillance for seasonal influenza provides a foundation to understand influenza seasonality, clinical disease burden, and economic impact. In tropical and subtropical climates, temporal trends for seasonal influenza are variable, and activity can be observed throughout the year [1, 2]. Southeast Asia hosts a variety of influenza circulation patterns, with countries maintaining one or two peaks of influenza and some reporting year‐round circulation [3, 4, 5]. Previous analyses of influenza surveillance data in Lao PDR have identified primary peaks of influenza around September and smaller secondary peaks around February during some years [1, 6, 7]. Strains of influenza A virus, including seasonal H3N2 and avian H5N1, often originate in Southeast Asia [8, 9], which underscores the need to maintain a strong surveillance capacity in the region. Influenza sentinel surveillance systems can provide valuable data for countries to understand local influenza circulation, detect novel strains, and inform strategies for pandemic preparedness and vaccine policy [10, 11, 12]. While epidemiologic information on influenza in Southeast Asia has expanded in recent years [5, 13, 14], data from some countries are still limited.

In 2006, Lao PDR developed the capacity to conduct real‐time reverse transcriptase polymerase chain reaction (RT‐PCR) testing for influenza virus at the National Center for Laboratory and Epidemiology (NCLE). An influenza‐like‐illness (ILI) sentinel surveillance system was established in the capital of Vientiane in 2007, and severe acute respiratory illness (SARI) surveillance began in 2008 [6, 7]. The surveillance system has since expanded to include hospitals in the northern and southern regions of the country. In 2010, NCLE was recognized as a National Influenza Center by WHO [7]. Analyses of the first few years of ILI and SARI surveillance data helped to inform the national influenza vaccination strategy in Lao PDR, which recommends vaccination with southern hemisphere vaccine for certain populations during May–July each year [6, 15]. Currently, influenza surveillance reports are produced weekly, and a comprehensive analysis of sentinel surveillance data from Lao PDR was conducted in 2010 [6]. However, influenza viruses constantly evolve, and the emergence of SARS‐CoV‐2 disrupted typical seasonal trends of influenza in some countries [16, 17, 18]. Continual monitoring and comprehensive analysis of influenza surveillance data are needed to inform vaccination policy and programming. This analysis reviews ILI and SARI sentinel surveillance data collected from 2016 to 2023 in nine sentinel hospitals in Lao PDR. We describe epidemiologic and virologic characteristics of influenza virus infection during this timeframe and the contribution of influenza‐associated ILI and SARI to hospital outpatient and inpatient loads.

## 2 Methods

### 2.1 Surveillance

**FIGURE 1:**
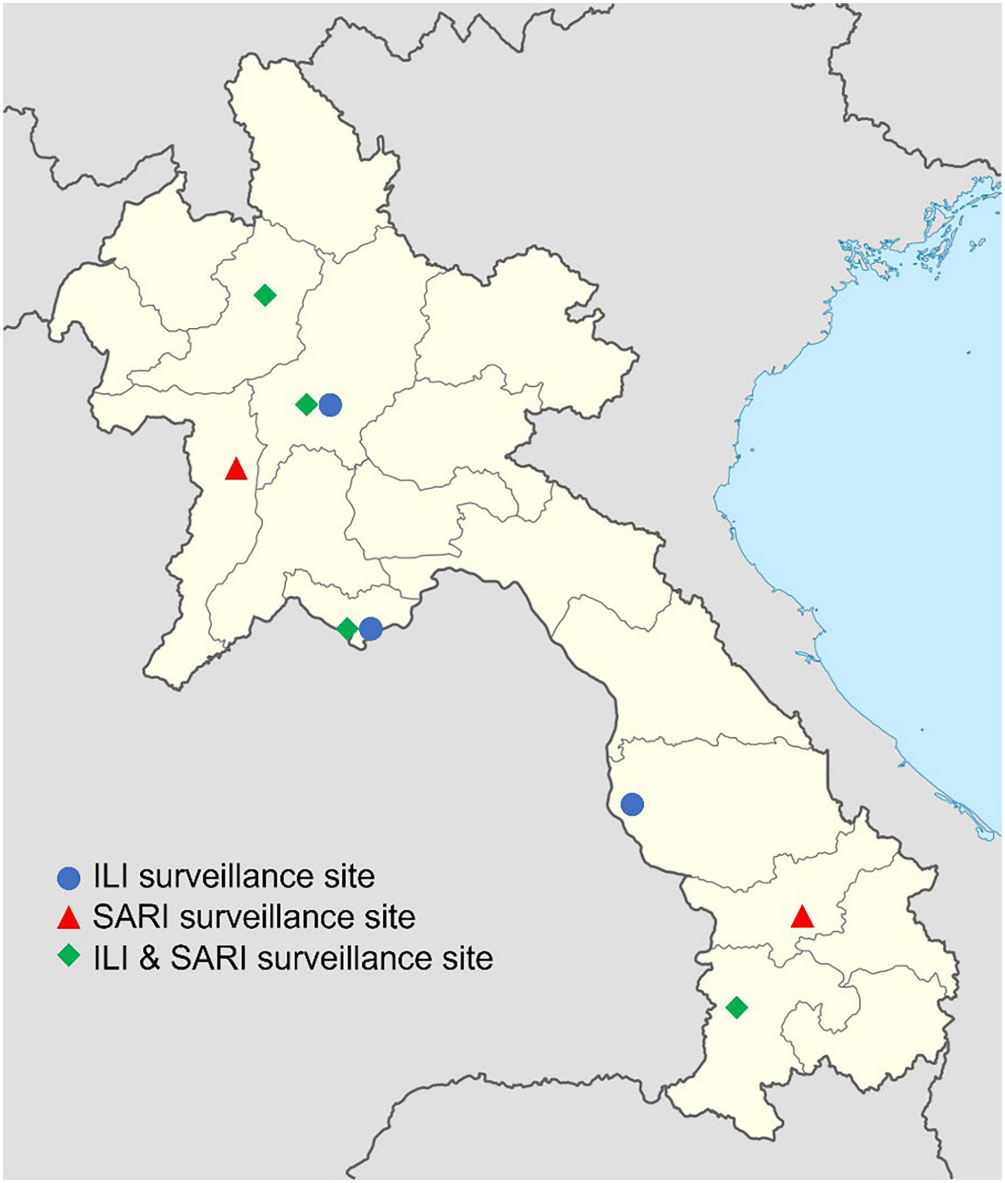
Location of influenza sentinel surveillance sites in Lao PDR, 2016–2023. Abbreviations: ILI, influenza‐like illness; PDR, People's Democratic Republic; SARI, severe acute respiratory illness.

Sentinel surveillance data on ILI and SARI were analyzed from January 2016 through December 2023 from nine hospitals in Lao PDR (Figure 1). ILI surveillance was conducted in outpatient departments in seven hospitals (three in the northern region, two in the central region, and two in the southern region), and SARI surveillance was conducted in inpatient departments in six hospitals (three in the northern region, one in the central region, and two in the southern region). This project was reviewed by the U.S. Centers for Disease Control and Prevention (CDC) and was conducted consistent with applicable federal law and CDC policy (e.g., 45 CFR 46.102(l) (2)).

For ILI surveillance, all patients reporting to outpatient departments were screened for respiratory symptoms and had their temperature measured and recorded. Illnesses that met the ILI case definition were recorded as ILI cases. All patients admitted to hospitals were screened for SARI using the same procedure at admission and every day during hospitalization. Aggregate numbers of ILI and SARI cases were recorded weekly.

The following modified WHO definitions [19] were used for screening ILI and SARI cases:

- ILI: acute respiratory infection with history of fever or measured fever of ≥ 38°C and cough and onset within the last 7 days.
- SARI: acute respiratory infection with history of fever or measured fever of ≥ 38°C and cough and onset within the last 7 days and required hospitalization.

Prior to 2018, specimens were collected daily in sentinel sites from all ILI and SARI cases. In practice, most specimens (85%) were collected Monday through Thursday. Beginning in 2018, sites were instructed to collect specimens on 1 day per week for each specimen type (ILI and SARI). From 2018 to 2023, most specimens (92%) were collected Monday through Wednesday. Specimens collected for influenza testing included one throat swab and either one nasopharyngeal swab or one nasal swab. A case report form was completed for ILI and SARI patients with a specimen collected. The form included information on patient demographics, illness onset, exposure to poultry, and self‐reported influenza vaccination status. Swabs were placed in viral transport media and transported on ice to the NCLE laboratory once weekly along with completed case report forms. To ensure quality, the total transport time was not to exceed 48 h. Specimens were tested by RT‐PCR for laboratory confirmation of influenza using primers and probes from the U.S. Centers for Disease Control and Prevention International Reagent Resource (IRR, formerly Influenza Reagent Resource) [20]. For influenza A–positive specimens, subtyping was performed, while lineage was determined for influenza B–positive specimens using additional real‐time PCR reagents from IRR. Untypable influenza specimens were referred to WHO Collaborating Centers for further testing. Specimens were also tested for human ribonuclease P (RNAseP) using a cycle threshold (Ct) cutoff of ≤ 40 to determine specimen quality.

### 2.2 Datasets

Data from case report forms were entered into a Pathogen Asset Control System database at NCLE and were linked to laboratory results. Additional data from each hospital's logbooks were entered into an Excel database and submitted weekly to NCLE. Data from outpatient department logbooks included total weekly outpatient visits by age group, total weekly ILI cases by age group, and total number of ILI specimens collected by age group. Data from inpatient department logbooks included total weekly numbers of hospital admissions by age and sex and the total weekly number of SARI cases by age and sex.

### 2.3 Data Analysis

Descriptive analyses were conducted on sentinel surveillance data. All analyses were conducted in SAS Version 9.4 (Cary, NC, United States). The numbers of ILI and SARI specimens tested and percent of specimens that were influenza positive were described by year, patient sex, patient age, region, and influenza vaccination status. Data were compared using chi‐square tests with a significance value of *p* < 0.05. Among positive specimens, the proportions of influenza A and B and of subtypes and lineages were described. Overall percent positivity was described by week of specimen collection, as well as by month and region for ILI and SARI specimens. Epidemiologic curves were constructed for the number of specimens positive for subtypes and lineages each month.

Proportional contributions of influenza‐associated ILI and SARI to outpatient and inpatient loads were estimated using WHO guidelines [21] for the years 2019–2023 (reliable aggregate counts for 2016–2018 were not available). First, we estimated the annual number of patients with influenza‐associated ILI by multiplying the percent positivity for ILI by the total number of patients with ILI for each week of the year and summing across all weeks. Next, we calculated the proportional contribution of influenza‐associated ILI to outpatient visits by dividing the number of influenza‐associated ILI visits by the total outpatient visits for each year. All calculations were conducted separately by age group. We followed the same procedures for SARI and inpatient visits. Hospital‐level estimates were combined nationally.

## 3 Results

A total of 31,533 specimens were submitted for influenza virus testing among nine sentinel surveillance sites during 2016–2023. On average, 75 specimens were tested for influenza weekly, of which 44 were from ILI patients and 31 were from SARI patients. The greatest number of specimens was tested in 2023 (5763 specimens), in the northern region (15,979 specimens), and among children aged < 2 years (11,189 specimens) (Table 1). Influenza vaccination status was unknown or missing for 64% of patients.

**TABLE 1:** Number of specimens tested, proportion positive for influenza, and distribution of influenza types by year, region, and demographic characteristics, Lao PDR, 2016–2023.

	No. tested	No. (%) influenza positive	*p* value^a^	No. (%) positive for influenza type, among positive specimens		
				Influenza A	Influenza B	*p* value^a^
Overall	31,533	4096 (13.0)		2760 (67.4)	1336 (32.6)	
Surveillance type			< 0.0001			0.0363
ILI	18,492	2761 (14.9)		1831 (66.3)	930 (33.7)	
SARI	13,041	1335 (10.2)		929 (69.6)	406 (30.4)	
Year			< 0.0001			< 0.0001
2016	4121	594 (14.4)		259 (43.6)	335 (56.4)	
2017	4505	692 (15.4)		464 (67.1)	228 (32.9)	
2018	3332	595 (17.9)		469 (78.8)	126 (21.2)	
2019	3204	476 (14.9)		250 (52.5)	226 (47.5)	
2020	3044	239 (7.9)		210 (87.9)	29 (12.1)	
2021	2570	107 (4.2)		107 (100.0)	0 (0.0)	
2022	4994	607 (12.2)		315 (51.9)	292 (48.1)	
2023	5763	786 (13.6)		686 (87.3)	100 (12.7)	
Region			< 0.0001			0.5960
North	15,979	1793 (11.2)		1199 (66.9)	230 (33.4)	
Central	3149	688 (21.8)		458 (66.6)	594 (33.1)	
South	12,405	1615 (13.0)		1103 (68.3)	512 (31.7)	
Sex			0.0048			0.2559
Male	16,400	2042 (12.5)		1393 (68.2)	649 (31.8)	
Female	15,133	2054 (13.6)		1367 (66.6)	687 (33.4)	
Age (years)			< 0.0001			< 0.0001
0–1	11,189	709 (6.3)		493 (69.5)	216 (30.5)	
2–4	5378	739 (13.7)		533 (72.1)	206 (27.9)	
5–14	4436	1092 (24.6)		680 (62.3)	412 (37.7)	
15–49	6929	1178 (17.0)		771 (65.4)	407 (34.6)	
50–64	2009	223 (11.1)		172 (77.1)	51 (22.9)	
≥ 65	1592	155 (9.7)		111 (71.6)	44 (28.4)	
Influenza vaccination status			< 0.0001			< 0.0001
Yes	275	37 (13.5)		29 (78.4)	8 (21.6)	
No	11,428	1577 (13.8)		972 (61.6)	605 (38.4)	
Do not know	18,611	2262 (12.2)		1601 (70.8)	661 (29.2)	
Missing data	1219	220 (18.0)		158 (71.8)	62 (28.2)	

Abbreviations: ILI, influenza‐like illness; PDR, People's Democratic Republic; SARI, severe acute respiratory illness.

^a^ Calculated using chi‐square tests.

Over 8 years of surveillance, the total positivity was 13.0% (Table 1). Influenza positivity was higher among ILI specimens compared with SARI specimens (14.9% vs. 10.2%, *p* < 0.0001). Influenza positivity over the 8‐year time period significantly differed by year, region, sex, and age. The highest influenza percent positivity was observed in 2018 (17.9%), in the central region (21.8%), and among individuals aged 5–14 years (24.6%). The lowest positivity rates were observed during 2020 and 2021 (7.9% and 4.2%, respectively) and among individuals aged < 2 (6.3%) and ≥ 65 (9.7%) years.

In all years except for 2021, cocirculation of influenza A and influenza B was observed (Table 1). Influenza A viruses accounted for 67.4% of all influenza‐positive specimens, and a slightly higher proportion of SARI was attributable to influenza A (69.6%) virus compared to ILI specimens (66.3%, *p* 0.0363). Proportions of influenza A and B viruses also differed by year (*p* < 0.0001) and age group (*p* < 0.0001).

The most commonly detected influenza virus subtypes were A/H1N1 (34.9% of positive specimens), A/H3 (32.3%), and B/Victoria (25.3%) (Table 2). The circulation of common subtypes differed by year; for example, in 2018, lower proportions of A/H3 (1.7%) and B/Victoria (0%) and higher proportions of A/pdmH1N1 (77.0%) and B/Yamagata (21.2%) were observed. Two cases of influenza A/H5 were detected during the 7 years. One case of human infection of avian influenza A/H5N1 was detected in 2020 in a 1‐year‐old girl, and one case of human infection of avian influenza A/H5N6 was detected in 2021 in a 5‐year‐old boy [22, 23]. Both cases were detected as a part of sentinel ILI and SARI surveillance.

**TABLE 2:** Proportion of influenza subtypes and lineages among positive specimens^a^, Lao PDR 2016–2023.

	A/pdmH1N1 *n* (%)	A/H3 *n* (%)	B Victoria *n* (%)	B Yamagata *n* (%)
Overall	1429 (34.9)	1325 (32.3)	1037 (25.3)	284 (6.9)
Surveillance type				
ILI	946 (34.3)	881 (31.9)	716 (25.9)	204 (7.4)
SARI	483 (36.2)	444 (33.3)	321 (24.0)	80 (6.0)
Year				
2016	168 (28.3)	91 (15.3)	304 (51.2)	25 (4.2)
2017	74 (10.7)	389 (56.2)	96 (13.9)	131 (18.9)
2018	458 (77.0)	10 (1.7)	0 (0.0)	126 (21.2)
2019	110 (23.1)	139 (29.2)	217 (45.6)	2 (0.4)
2020	59 (24.7)	149 (62.3)	28 (11.7)	0 (0.0)
2021	0 (0.0)	106 (99.1)	0 (0.0)	0 (0.0)
2022	2 (0.3)	313 (51.6)	292 (48.1)	0 (0.0)
2023	558 (71.0)	128 (16.3)	100 (12.7)	0 (0.0)
Region				
North	596 (33.2)	601 (33.5)	472 (26.3)	118 (6.6)
Central	192 (27.9)	266 (38.7)	166 (24.1)	61 (8.9)
South	641 (39.7)	458 (28.4)	399 (24.7)	105 (6.5)
Sex				
Male	710 (34.8)	680 (33.3)	510 (25.0)	134 (6.6)
Female	719 (35.0)	645 (31.4)	527 (25.7)	150 (7.3)
Age (years)				
0–1	251 (35.4)	240 (33.9)	180 (25.4)	35 (4.9)
2–4	307 (41.5)	224 (30.3)	164 (22.2)	37 (5.0)
5–14	384 (35.2)	294 (26.9)	314 (28.8)	95 (8.7)
15–49	359 (30.5)	412 (35.0)	326 (27.7)	75 (6.4)
50–64	89 (39.9)	83 (37.2)	25 (11.2)	26 (11.7)
≥ 65	39 (25.2)	72 (46.5)	28 (18.1)	16 (10.3)

Abbreviations: ILI, influenza‐like illness; PDR, People's Democratic Republic; SARI, severe acute respiratory illness.

^a^ Not shown are influenza A untypable (*n* = 3), influenza A/H1 N untypable (*n* = 1), influenza A/H5 (*n* = 2), and influenza B uncharacterized (*n* = 15).

**FIGURE 2:**
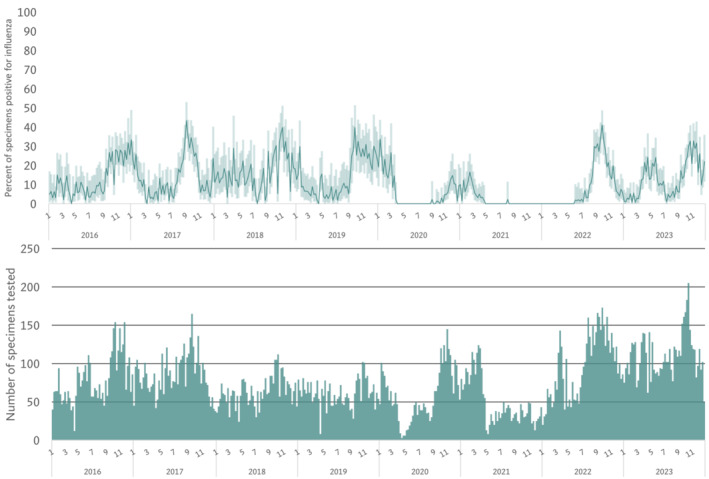
Weekly percent positivity for influenza (top) and number of ILI and SARI specimens tested for influenza (bottom), Lao PDR 2016–2023. Abbreviations: PDR, People's Democratic Republic.

Detection of influenza among patients with ILI and SARI occurred throughout the year, but overall, influenza activity peaked from September through January (Figure 2 and Figures S1 and S2). In some years (e.g., 2018 and 2023), increased influenza positivity was observed from January through May. Weekly positivity for ILI and SARI is also shown separately (Figure S1). Overall, the temporal trends for influenza positivity are similar for ILI and SARI. Influenza peak activity in the southern region appeared to precede the northern region in most years by about 1 month (Figure S2).

**FIGURE 3:**
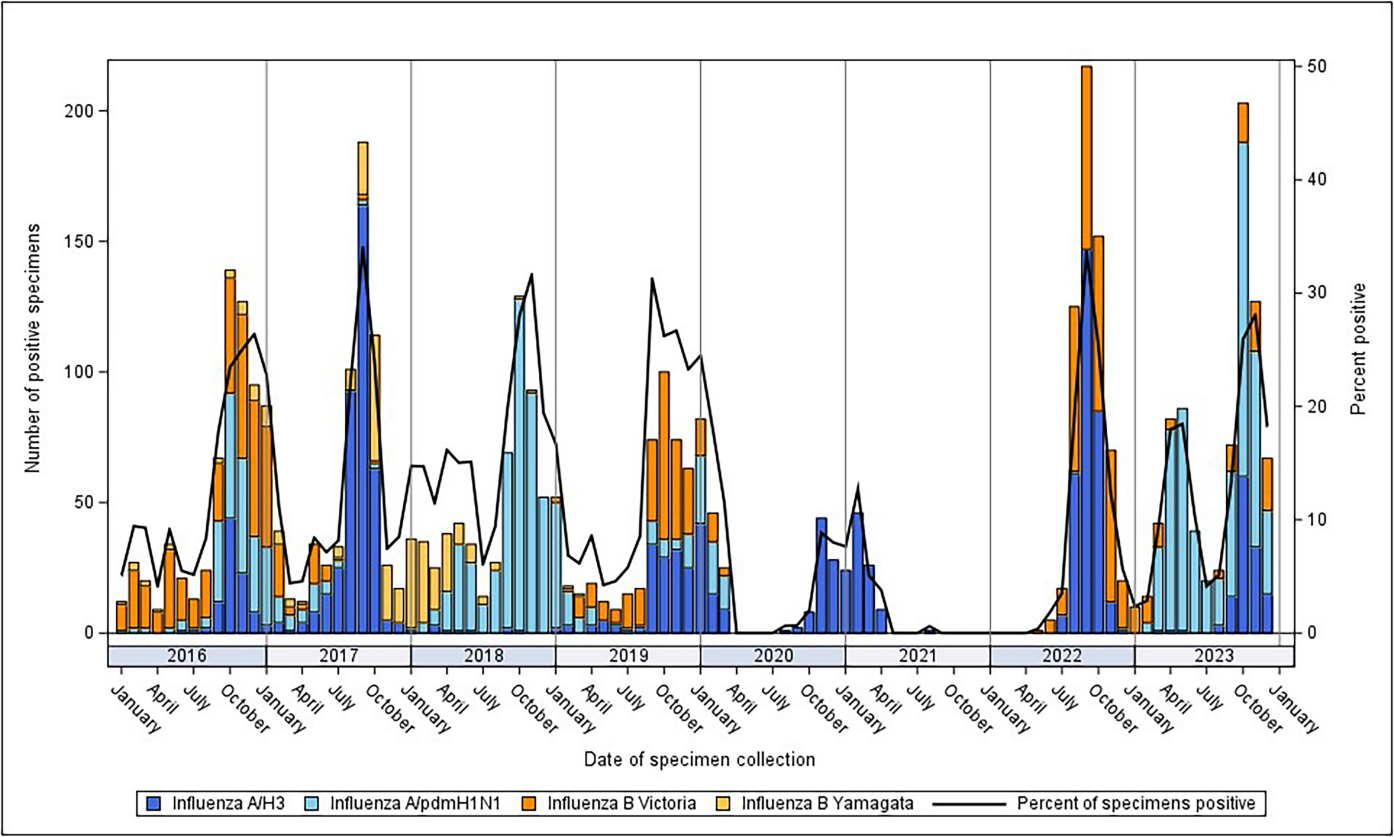
Proportion of influenza subtypes detected by month, Lao PDR 2016–2023. Abbreviations: PDR, People's Democratic Republic.

Nationally, influenza A (A/H3 or A/pdmH1N1) peaked annually, with the exception of 2021 (Figure 3). During most years with high A/H3 activity, fewer specimens were positive for A/pdmH1N1, and vice versa. Influenza B/Victoria peaked every 3 years, in 2016, 2019, and 2022. The highest numbers of specimens positive for B/Yamagata occurred in the 2017–2018 season. The last B/Yamagata positive specimen was collected on 19 March 2019. At a regional level, influenza virus subtypes and lineages detected over time were similar by region (Figure S3).

**FIGURE 4:**
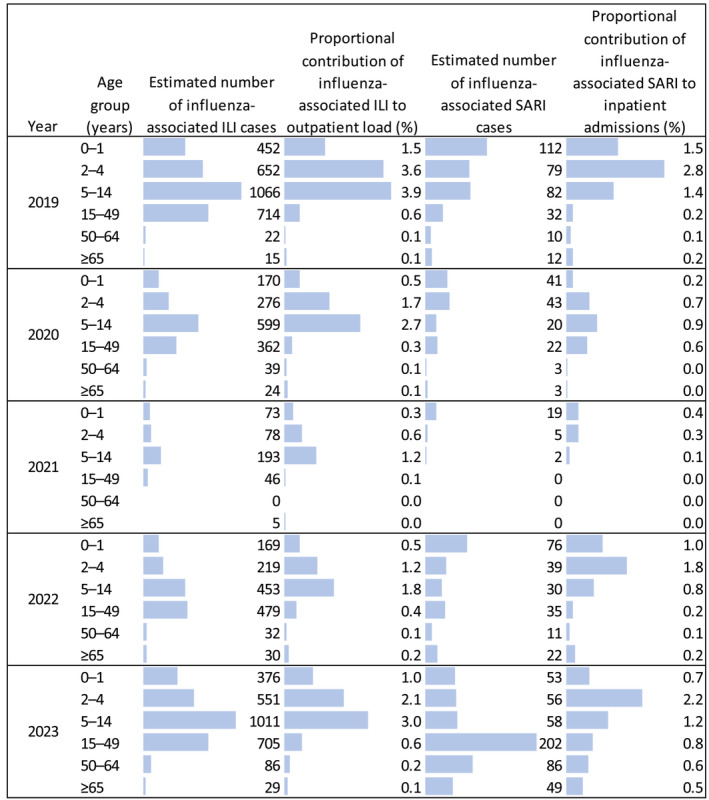
Proportional contributions of influenza‐associated ILI and SARI to hospital loads at nine sentinel sites, Lao PDR 2019–2023. ILI, influenza‐like illness; PDR, People's Democratic Republic; SARI, severe acute respiratory illness.

Estimated proportional contributions of influenza‐associated ILI and SARI to outpatient and inpatient loads by age category are shown in Figure 4. The proportional contribution of influenza‐associated ILI and SARI was highest among younger age groups (in patients aged 0–14 years). In 2019, influenza‐associated illnesses contributed to 3.9% of outpatient visits for children aged 5–14 years and 2.8% of inpatient hospitalizations for young children aged 2–4 years. Among the 15–49, 50–64, and ≥ 65 age groups during the same year, proportional contributions of influenza to outpatient and inpatient loads were estimated to be less than 1%. In the years 2020 and 2021, contributions of influenza‐associated ILI and SARI to outpatient and inpatient loads decreased, but these proportions rose again in 2022 and 2023.

## 4 Discussion

From 2016 to 2023, influenza was detected in a substantial proportion of ILI and SARI specimens in Lao PDR. Since 2006, consistent influenza surveillance and laboratory reporting across the country has provided clinicians and public health authorities with reliable information on geographic and temporal patterns of influenza transmission. NCLE summarizes influenza surveillance data and communicates findings in a weekly report to hospitals, public health authorities, and partners. NCLE and partners use these data to monitor the occurrence and potential spread of outbreaks, to detect any abnormal events or patterns, and to inform vaccination programs. The current analysis provides a comprehensive view of recent influenza surveillance data in Lao PDR and can inform improvements to the surveillance system and influenza vaccination policy and timing.

In response to COVID‐19 in April 2020, Lao PDR implemented a 2‐month lockdown with stay‐at‐home orders and business and school closings [24]. Restrictions on mass gatherings, limitations on travel, implementation of curfews, and encouragement of social distancing, mask wearing, and other personal protective measures continued intermittently during 2020 and 2021 [25, 26]. Influenza activity declined during the 2 years after the emergence of SARS‐CoV‐2, then increased in 2022, and returned to pre‐COVID‐19 levels, coinciding with lifting of COVID‐19 restrictions [24, 26]. Analyses in other countries have reported similar observations following the emergence of SARS‐CoV‐2 [27, 28, 29]. In some countries, like Senegal, influenza activity after the emergence of SARS‐CoV‐2 occurred outside of the expected transmission season [18]. In contrast, our results from surveillance in Lao PDR suggest that influenza peak activity resumed its typical pattern with a peak in September 2022 and October 2023. These findings of fluctuating influenza positivity in recent years emphasize the importance of maintaining strong respiratory virus surveillance systems as SARS‐CoV‐2 and influenza, and other respiratory viral pathogens continue to cocirculate.

Our results showed that influenza virus is detected year‐round in Lao PDR with positivity increasing in August and September, peaking during September through January, and occurring approximately 1 month earlier in the south than the north in most years. Because it takes approximately 2 weeks after influenza vaccination to develop protective immunity and because vaccine effectiveness wanes in the year following vaccination, it is important to monitor peak influenza activity and time influenza vaccination campaigns accordingly [30]. The findings from this analysis suggest that the current practice of conducting influenza vaccination campaigns during May–July in Lao PDR is reasonable, in anticipation of increasing influenza cases during August and September [5, 15]. Further analysis to establish seasonal and alert thresholds is an important next step for Lao PDR. In Cambodia, influenza thresholds have been used to improve communication with the medical community and general public [31]. Thresholds in Lao PDR could be assessed weekly and would provide public health practitioners and clinicians with a consistent measurement of circulating influenza activity.

An earlier analysis of ILI surveillance data in Lao PDR during 2008–2011 reported increased influenza activity from July through December [6], consistent with our findings. An increase in the number of specimens tested monthly in our current report (> 300 per month) compared with the previous report (< 100 per month) provides stable estimates to confirm the timing of peak influenza activity. Influenza virus circulation in Lao PDR peaks slightly later in comparison with Thailand (August–September) and Cambodia (June–December) and differs from Viet Nam (March–July) despite geographic proximity [31, 32, 33, 34]. As our report has demonstrated, the timing of peak influenza activity varies slightly at the subnational level, which makes it difficult to compare across countries at the national level. This regional variability emphasizes the importance of designing the surveillance system to collect an appropriate number of specimens from geographically representative sites to monitor influenza circulation at national and subnational levels.

In contrast to the timing of peak positivity, detection of influenza virus subtypes and lineages did not appreciably differ by region. This suggests that the number of sentinel surveillance sites needed for a national influenza sentinel surveillance system could be the minimum number of sites needed to reach a target national weekly sample size (based on achieving surveillance objectives) and geographic representation. Given the similarity in influenza virus subtype and lineage across regions, adding additional sentinel surveillance sites might add additional costs without substantially improving insight into influenza circulation. Lao PDR is using the findings from this analysis to re‐evaluate the number of sentinel sites needed in the country to achieve its surveillance objectives and potentially reduce surveillance costs.

We found that influenza has a substantial impact on children in Lao PDR. Among children ages 5–14 years with ILI, approximately one in four tested positive for influenza. Influenza‐associated ILI had the highest contribution to outpatient visits for children 5–14 years of age (approximately 1 in 25 outpatient visits in this age group was for influenza‐associated ILI). These findings are consistent with a prospective household cohort study conducted in Lao PDR in 2015–2016. This study found that influenza A incidence was highest for children ages 5–14 years, and influenza B incidence was highest for children < 15 years of age [35]. For more severe infections, we found that the contribution of influenza‐associated SARI to inpatient hospitalizations was highest for children ages 2–4 years. In absolute measures, the highest number of influenza‐associated SARI cases in most years occurred among infants < 2 years of age.

In Lao PDR, antiviral treatment for influenza is not widely available. Vaccination is the best preventive measure against influenza. Lao PDR has had a national vaccination program since 2012 and has recently worked to integrate influenza and COVID‐19 into a single national vaccination policy [15]. Populations recommended for influenza vaccination include health care workers, pregnant women, children 6–59 months, adults 60 years and older, and people with chronic diseases. Studies in Lao PDR have demonstrated that vaccinating health care workers is cost‐saving and vaccinating pregnant women and adults 60 years and older against influenza is cost effective [36]. However, vaccine supply is limited, and the Lao Ministry of Health must decide how to prioritize influenza vaccinations. Additional studies to estimate population‐level burden of influenza have complemented influenza surveillance but are costly and time consuming to conduct frequently. An influenza burden analysis in Lao PDR with data from 2016 identified youngest (< 5 years) and oldest (≥ 65 years) age groups as having the highest burden of influenza hospitalizations [37]. Our report found that influenza continues to be an important contributor to SARI hospitalizations, particularly for children < 5 years.

Our analysis is subject to several limitations. The sampling method used a modified convenience sampling approach based on sampling 1 day per week toward the beginning of each week. This approach could introduce biases by excluding patients who preferentially access care during evenings, weekends, or later in the week. Specimens collected outside of standardized procedures (e.g., on weekend days) could be influenced by health provider judgement, which might affect the percent positivity. The inclusion of central and provincial hospitals rather than district level hospitals and primary health centers might exclude patients with less severe illness and those living in rural areas. Finally, more limited specimen collection in the central region made it difficult to identify temporal trends in this region. Some modifications to influenza surveillance could improve the usefulness of influenza surveillance. These include incorporating data elements to capture illness severity (e.g., intensive care unit admission and invasive mechanical ventilation) among SARI cases. Use of a national immunization registry or vaccination card for all ages would help to improve data quality and completeness for vaccination history. Existing knowledge of catchment areas for sentinel sites could be used to calculate incidence rates by influenza virus type and subtype [37]. Understanding vaccination coverage for recommended vaccination groups would help to identify vaccination coverage gaps, inform vaccination outreach and communication strategies, and potentially allow Lao PDR to calculate vaccine effectiveness.

## 5 Conclusions

ILI and SARI surveillance in Lao PDR continues to improve our understanding of influenza incidence, temporal trends, and subtype circulation and allows Lao Ministry of Health to detect and respond to human cases of avian influenza. Training for epidemiology and laboratory staff to monitor respiratory viral pathogens helps to maintain public health preparedness for responding to emerging respiratory threats with pandemic potential. Influenza surveillance data allow Lao PDR to continually evaluate priority populations for vaccination and identify the optimal timing of influenza vaccination campaigns. Because influenza vaccine‐derived immunity wanes over time, vaccines with more durable immunity would be of great benefit to countries like Lao PDR where influenza transmission occurs year‐round [38]. Improving data quality of vaccination history and adding data elements on severity (e.g., ICU admission and mechanical ventilation) would further improve the usefulness of surveillance. Surveillance for respiratory viral pathogens is important to maintain as influenza virus and SARS‐CoV‐2, and other respiratory viral pathogens continue to cocirculate.

## Author Contributions

**Natalie Wodniak:** conceptualization, data curation, formal analysis, project administration, visualization, writing–original draft, writing–review and editing, methodology. **KeoOudomphone Vilivong:** investigation, project administration, validation, writing–review and editing. **Bouaphanh Khamphaphongphane:** conceptualization, investigation, project administration, supervision, writing–review and editing, validation. **Bounthanom Sengkeopraseuth:** conceptualization, investigation, project administration, supervision, writing–review and editing, validation. **Virasack Somoulay:** conceptualization, investigation, project administration, supervision, validation, writing–review and editing. **May Chiew:** writing–review and editing. **Pakapak Ketmayoon:** writing–review and editing. **Melissa Jiao:** writing–review and editing. **Sonesavanh Phimmasine:** writing–review and editing. **Kim Carmela Co:** writing–review and editing. **Phetdavanh Leuangvilay:** writing–review and editing. **Satoko Otsu:** conceptualization, funding acquisition, project administration, resources, supervision, writing–review and editing. **Viengphone Khanthamaly:** investigation, project administration, writing–review and editing. **Phayvanh Keopaseuth:** conceptualization, supervision, writing–review and editing. **William W. Davis:** conceptualization, methodology, project administration, resources, supervision, writing–review and editing. **Martha P. Montgomery:** conceptualization, data curation, formal analysis, methodology, supervision, visualization, writing–original draft, writing–review and editing. **Phonepadith Xangsayyarath:** conceptualization, investigation, project administration, supervision, validation, writing–review and editing.

## Disclosure

The findings and conclusions in this article are those of the authors and do not necessarily represent the official position of the U.S. Centers for Disease Control and Prevention (CDC).

## Ethics Statement

This project was reviewed by the Centers for Disease Control and Prevention (CDC) and was conducted consistent with applicable federal law and CDC policy (e.g., 45 CFR 46.102(l) (2)).

## Conflicts of Interest

May Chiew, Pakapak Ketmayoon, Melissa Jiao, Sonesavanh Phimmasine, Kim Carmela Co, Phetdavanh Leuangvilay, and Satoko Otsu reported receiving financial support from U.S. Centers for Disease Control and Prevention and U.S. Defense Threat Reduction Agency for attending meetings and for official travel.

### Peer Review

The peer review history for this article is available at https://www.webofscience.com/api/gateway/wos/peer‐review/10.1111/irv.13353.

## Supporting information


**Figure S1.** Weekly percent of ILI and SARI specimens positive for influenza, Lao PDR 2016–2023.
**Figure S2.** Monthly influenza percent positivity among ILI and SARI specimens by region†, Lao PDR 2016–2023. †Central region not shown due to fewer specimens collected and consequently unstable estimates.
**Figure S3.** Number of influenza subtypes and lineages detected by region, Lao PDR 2016–2023.

## Data Availability

Data is available on request from the authors.
