# Supplementary material for: Epidemiologic and Virologic Characteristics of Influenza in Lao PDR, 2016–2023
Source: Influenza Other Respir Viruses. 2024 Aug 5;18(8):e13353. doi: 10.1111/irv.13353 (PMC11300510; doi:10.1111/irv.13353)
Supplement: Supplementary file 1 — Figure S1. Weekly percent of ILI and SARI specimens positive for influenza, Lao PDR 2016–2023. Figure S2. Monthly influenza percent positivity among ILI and SARI specimens by region†, Lao PDR 2016–2023. †Central region not shown due to fewer specimens collected and consequently unstable estimates. Figure S3. Number of influenza subtypes and lineages detected by region, Lao PDR 2016–2023. [file IRV-18-e13353-s001.docx]

Supplemental Figure 1. Weekly percent of ILI and SARI specimens positive for influenza, Lao PDR 2016–2023


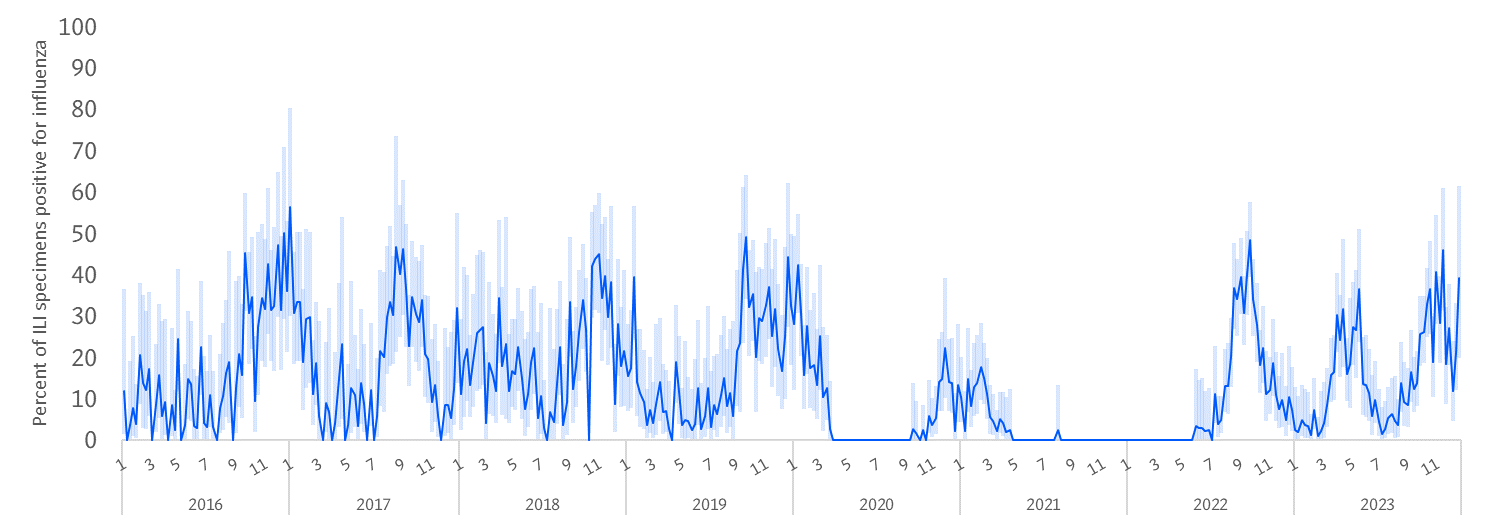

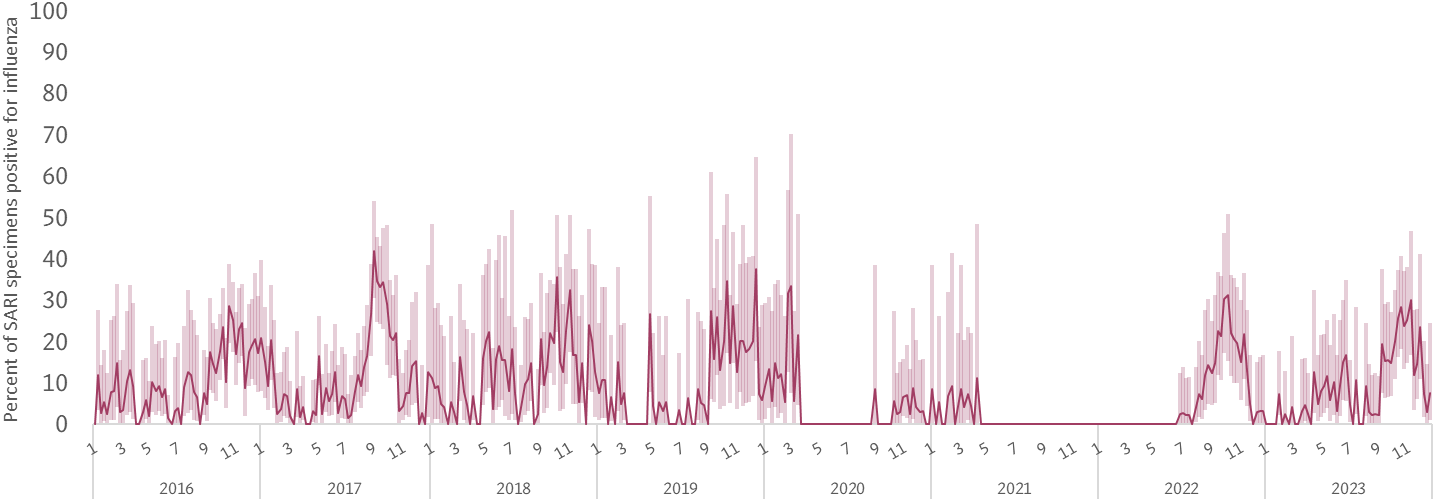


Supplemental Figure 2. Monthly influenza percent positivity among ILI and SARI specimens by region†, Lao PDR 2016–2023


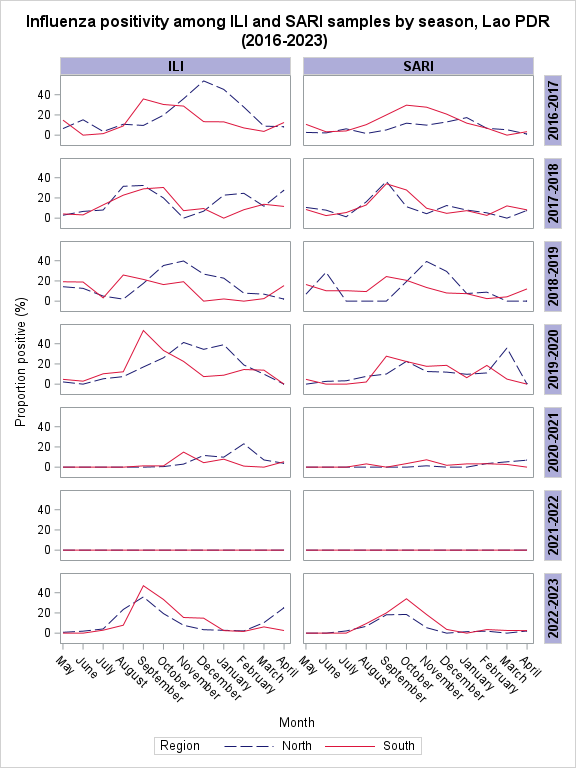


†Central region not shown due to fewer specimens collected and consequently unstable estimates.

Supplemental Figure 3. Number of influenza subtypes and lineages detected by region, Lao PDR 2016–2023**
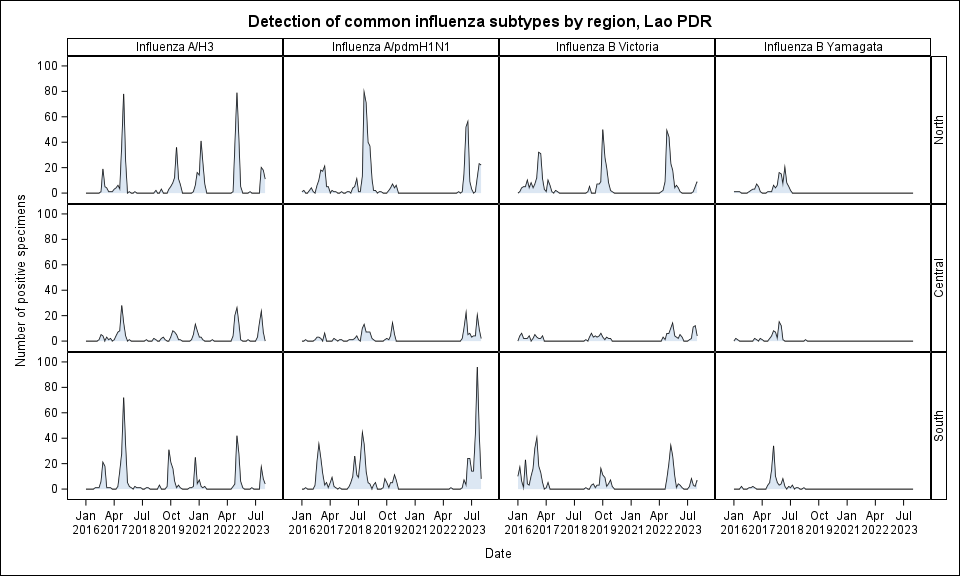
**
